# *tert*-Butyl 4-carbamoyl-3-methoxy­imino-4-methyl­piperidine-1-carboxyl­ate

**DOI:** 10.1107/S1600536808036106

**Published:** 2008-11-08

**Authors:** Juxian Wang, Mingliang Liu, Jue Cao, Yucheng Wang

**Affiliations:** a Institute of Medicinal Biotechnology, Chinese Academy of Medical Sciences and Perking Union Medical College, Beijing 100050, People’s Republic of China

## Abstract

The title compound, C_13_H_23_N_3_O_4_, was prepared starting from ethyl *N*-benzyl-3-oxopiperidine-4-carboxyl­ate through a nine-step reaction, including hydrogenation, Boc (*tert*-butoxy­carbon­yl) protection, methyl­ation, oximation, hydrolysis, esterification and ammonolysis. In the crystal structure, mol­ecules are linked by inter­molecular N—H⋯O hydrogen bonds to form a porous three-dimensional network with solvent-free hydro­phobic channels extending along the *c* axis.

## Related literature

For the synthesis and properties of quinolone derivatives, see: Ray *et al.* (2005 ▶); Ball *et al.* (1998 ▶); Bryskier (1997 ▶); De Sarro & De Sarro (2001 ▶); Anderson & Osheroff (2001 ▶); Dang *et al.* (2007 ▶); Wang *et al.* (2008 ▶).
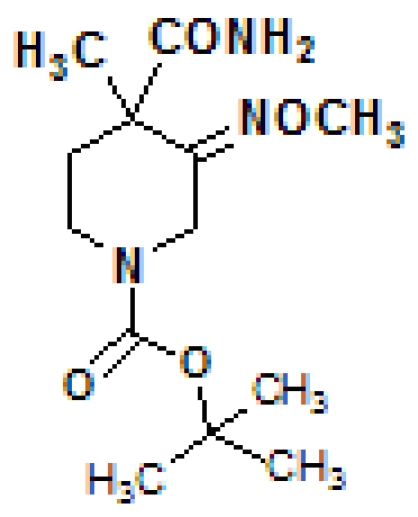


## Experimental

### 

#### Crystal data

C_13_H_23_N_3_O_4_
*M* *_r_* = 285.34
Tetragonal, 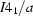

*a* = 22.813 (2) Å
*c* = 12.0742 (16) Å
*V* = 6283.8 (11) Å^3^
*Z* = 16
Mo *K*α radiation
μ = 0.09 mm^−1^
*T* = 293 (2) K
0.48 × 0.46 × 0.45 mm

#### Data collection

Bruker SMART APEX CCD diffractometer
Absorption correction: multi-scan (*SADABS*; Sheldrick, 1996 ▶) *T*_min_ = 0.957, *T*_max_ = 0.963
16003 measured reflections
2763 independent reflections
1794 reflections with *I* > 2σ(*I*)
*R*_int_ = 0.049

#### Refinement

*R*[*F* ^2^ > 2σ(*F* ^2^)] = 0.039
*wR*(*F* ^2^) = 0.113
*S* = 1.01
2763 reflections
181 parameters
H-atom parameters constrained
Δρ_max_ = 0.19 e Å^−3^
Δρ_min_ = −0.12 e Å^−3^

### 

Data collection: *SMART* (Bruker, 1998 ▶); cell refinement: *SAINT* (Bruker, 1999 ▶); data reduction: *SAINT*; program(s) used to solve structure: *SHELXS97* (Sheldrick, 2008 ▶); program(s) used to refine structure: *SHELXL97* (Sheldrick, 2008 ▶); molecular graphics: *SHELXTL* (Sheldrick, 2008 ▶); software used to prepare material for publication: *SHELXTL*.

## Supplementary Material

Crystal structure: contains datablocks global, I. DOI: 10.1107/S1600536808036106/rz2260sup1.cif

Structure factors: contains datablocks I. DOI: 10.1107/S1600536808036106/rz2260Isup2.hkl

Additional supplementary materials:  crystallographic information; 3D view; checkCIF report

## Figures and Tables

**Table 1:** Hydrogen-bond geometry (Å, °)

*D*—H⋯*A*	*D*—H	H⋯*A*	*D*⋯*A*	*D*—H⋯*A*
N3—H3*A*⋯O4^i^	0.86	2.11	2.9607 (18)	173
N3—H3*B*⋯O3^ii^	0.86	2.34	3.1334 (19)	153

Symmetry codes: (i) 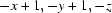; (ii) 
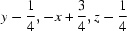
.

**Fig. 1.**
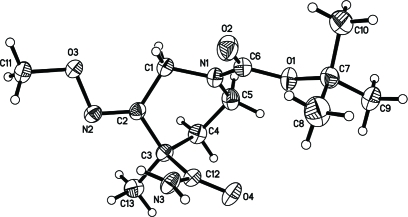
The molecular structure of the title compound, showing 40% probability displacement ellipsoids and the atom-numbering scheme.

**Fig. 2.**
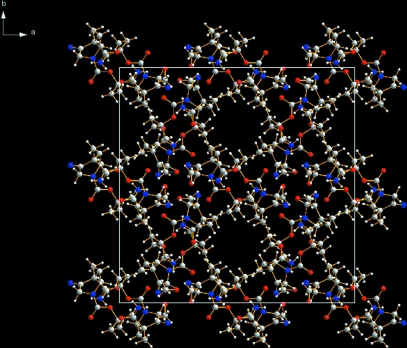
Crystal packing of the title compound viewed along the c axis.

## supplementary crystallographic information

### Comment

Quinolones, a class of synthetic antibacterial compounds based on a 4-quinolone skeleton, have been the landmark discovery in the treatment of bacterial infections in both community and hospital setting (Ray *et al.*, 2005; Ball *et al.*, 1998; Bryskier, 1997). The most intensive structural variations have been carried out on the basic group at the C-7 position, partially due to the ease of their introduction through a nucleophilic aromatic substitution reaction on the corresponding halide. Piperazine, aminopyrolidine and their derivatives have been the most successfully employed side chains, as evidenced by the compounds currently on the market (De Sarro & De Sarro, 2001; Anderson & Osheroff, 2001; Dang *et al.*, 2007). Recently, as part of an ongoing study aimed to find potent and broad-spectrum antibacterial agents displaying strong Gram-positive activity, we have focused our attention on the synthesis of C-7 substituted quinolones (Wang *et al.*, 2008). We report here the crystal structure of the title compound, which is a key intermediate of 3-methoxyimino-4-amino-4-methylpiperidine.

In the molecule of the title compound (Fig. 1), the N1—C6 (1.352 (2) Å) and N3—C12 (1.323 (2) Å) bond lengths are significantly shorter than the normal C—N single bond (1.47 Å), indicating some conjugation with the C6═O2 and C12═O4 carbonyl groups, respectively. The six-membered piperidine ring adopts a boat conformation, with N1 and C3 displaced by 0.533 (2) and 0.632 (2) Å, respectively, from the mean-plane through C1, C2, C4 and C5. In the crystal structure, molecules are linked by intermolecular N—H···O hydrogen bonds (Table 1) to form a porous three-dimensional network with solvent-free hydrophobic channels extending along the *c* axis (Fig. 2).

### Experimental

To a solution of ethyl *N*-Boc-3-methoxyimino-4-methylpiperidine-4-carboxylate (12.71 g, 40.5 mmol) in ethanol (50 ml) was added dropwise a solution of sodium hydroxide (2.75 g, 68.85 mmol) in water (5 ml) at room temperature. After stirring for 4.5 h, ethanol was removed under reduced pressure. After addition of water (20 ml), acetic acid (5 ml, 86.5 mmol) and triethylamine (17 ml, 122 mmol), the mixture was stirred for 10 min and extracted with CH_2_Cl_2_ (3 × 40 ml). The combined organic extracts were washed with saturated brine (3 × 20 ml) and dried over anhydrous sodium sulfate. The reaction mixture was then cooled to 259-261 K, and isobutyl chloroformate (13.1 ml, 101.8 mmol) was added. After 0.5 h, the reaction mixture was washed with 1 N HCl (4 × 20 ml) and saturated brine (4 × 40 ml), and dried over anhydrous sodium sulfate. The resulting yellow residue was purified by column chromatography, with petroleum ether/diethyl ether (3:1 v/v) as eluent to afford the title compound (4.92 g, 42.6%; mp: 140–142 °C). Single crystals suitable for X-ray analysis were obtained by slow evaporation of an ethanol/ethyl acetoacetate solution (1:1 v/v). ^1^H NMR (CDCl_3_, δ): 1.37-1.46 (12H, m, CH_3_), 1.50-1.57 (1H, m, C_5_), 2.43-2.49 (1H, m, C_5_), 3.38-3.53 (2H, m, C_6_), 3.89 (3H, s, OCH_3_), 4.17-4.45 (2H, m, C_2_), 5.57 (1H, br, CONH), 6.00 (1H, br, CONH). MS (ESI, m/z): 286 (M+1)^+^.

### Refinement

All H atoms were placed at calculated positions, with C—H = 0.95–0.98 Å, N—H = 0.86 Å, and included in the final cycles of refinement using a riding model, with *U*_iso_(H) = 1.2 *U*_eq_(C, N) or 1.5 *U*_eq_(C) for methyl H atoms. The crystal structure contains voids of about 105 Å^3^ connected to form channels along the *c* axis, which may accommodate solvent molecules. However, significant residual densities in the void could not be observed in the difference Fourier map.

### Crystal data

**Table:**

C_13_H_23_N_3_O_4_	*Z* = 16
*M**_r_* = 285.34	*F*_000_ = 2464
Tetragonal, *I*4_1_/*a*	*D*_x_ = 1.206 Mg m^−^^3^
Hall symbol: -I 4ad	Mo *K*α radiation λ = 0.71073 Å
*a* = 22.813 (2) Å	Cell parameters from 3595 reflections
*b* = 22.813 (2) Å	θ = 2.5–22.6º
*c* = 12.0742 (16) Å	µ = 0.09 mm^−^^1^
α = 90º	*T* = 293 (2) K
β = 90º	Block, colorless
γ = 90º	0.48 × 0.46 × 0.45 mm
*V* = 6283.8 (11) Å^3^	

### Data collection

**Table:**

Bruker SMART APEX CCD diffractometer	2763 independent reflections
Radiation source: fine-focus sealed tube	1794 reflections with *I* > 2σ(*I*)
Monochromator: graphite	*R*_int_ = 0.049
*T* = 293(2) K	θ_max_ = 25.0º
φ and ω scans	θ_min_ = 1.8º
Absorption correction: multi-scan(SADABS; Sheldrick, 1996)	*h* = −27→27
*T*_min_ = 0.957, *T*_max_ = 0.963	*k* = −27→18
16003 measured reflections	*l* = −14→14

### Refinement

**Table:**

Refinement on *F*^2^	Secondary atom site location: difference Fourier map
Least-squares matrix: full	Hydrogen site location: inferred from neighbouring sites
*R*[*F*^2^ > 2σ(*F*^2^)] = 0.039	H-atom parameters constrained
*wR*(*F*^2^) = 0.113	*w* = 1/[σ^2^(*F*_o_^2^) + (0.0607*P*)^2^] where *P* = (*F*_o_^2^ + 2*F*_c_^2^)/3
*S* = 1.01	(Δ/σ)_max_ < 0.001
2763 reflections	Δρ_max_ = 0.19 e Å^−^^3^
181 parameters	Δρ_min_ = −0.12 e Å^−^^3^
Primary atom site location: structure-invariant direct methods	Extinction correction: none

### Special details

**Table:**

Geometry. All e.s.d.'s (except the e.s.d. in the dihedral angle between two l.s. planes) are estimated using the full covariance matrix. The cell e.s.d.'s are taken into account individually in the estimation of e.s.d.'s in distances, angles and torsion angles; correlations between e.s.d.'s in cell parameters are only used when they are defined by crystal symmetry. An approximate (isotropic) treatment of cell e.s.d.'s is used for estimating e.s.d.'s involving l.s. planes.
Refinement. Refinement of *F*^2^ against ALL reflections. The weighted *R*-factor *wR* and goodness of fit *S* are based on *F*^2^, conventional *R*-factors *R* are based on *F*, with *F* set to zero for negative *F*^2^. The threshold expression of *F*^2^ > σ(*F*^2^) is used only for calculating *R*-factors(gt) *etc*. and is not relevant to the choice of reflections for refinement. *R*-factors based on *F*^2^ are statistically about twice as large as those based on *F*, and *R*- factors based on ALL data will be even larger.

### Fractional atomic coordinates and isotropic or equivalent isotropic displacement parameters (Å^2^)

**Table:**

	*x*	*y*	*z*	*U*_iso_*/*U*_eq_	
N1	0.38925 (6)	0.53733 (7)	0.27764 (13)	0.0504 (4)	
N2	0.29390 (6)	0.58484 (6)	0.05570 (12)	0.0430 (4)	
N3	0.41951 (6)	0.51996 (6)	−0.00791 (13)	0.0484 (4)	
H3A	0.4432	0.4941	−0.0342	0.058*	
H3B	0.3830	0.5189	−0.0253	0.058*	
O1	0.46173 (5)	0.48101 (5)	0.33671 (11)	0.0554 (4)	
O2	0.37946 (6)	0.43856 (6)	0.26866 (13)	0.0694 (4)	
O3	0.24359 (5)	0.55589 (5)	0.09853 (10)	0.0501 (4)	
O4	0.49117 (5)	0.56448 (5)	0.08658 (12)	0.0570 (4)	
C1	0.33172 (8)	0.54686 (10)	0.23109 (17)	0.0558 (5)	
H1A	0.3080	0.5692	0.2828	0.067*	
H1B	0.3126	0.5094	0.2189	0.067*	
C2	0.33613 (7)	0.57937 (7)	0.12372 (14)	0.0400 (4)	
C3	0.39477 (7)	0.60751 (7)	0.09840 (14)	0.0405 (4)	
C4	0.41585 (8)	0.63467 (8)	0.20750 (15)	0.0502 (5)	
H4A	0.4531	0.6541	0.1949	0.060*	
H4B	0.3879	0.6642	0.2309	0.060*	
C5	0.42325 (9)	0.58985 (8)	0.30031 (16)	0.0558 (5)	
H5A	0.4643	0.5796	0.3074	0.067*	
H5B	0.4105	0.6069	0.3698	0.067*	
C6	0.40799 (8)	0.48163 (10)	0.29224 (16)	0.0503 (5)	
C7	0.49544 (8)	0.42673 (9)	0.34839 (16)	0.0544 (5)	
C8	0.50169 (11)	0.39737 (11)	0.2370 (2)	0.0890 (8)	
H8A	0.4643	0.3824	0.2138	0.134*	
H8B	0.5292	0.3657	0.2426	0.134*	
H8C	0.5155	0.4254	0.1837	0.134*	
C9	0.55329 (9)	0.44949 (10)	0.3911 (2)	0.0709 (6)	
H9A	0.5703	0.4754	0.3372	0.106*	
H9B	0.5794	0.4172	0.4041	0.106*	
H9C	0.5471	0.4704	0.4590	0.106*	
C10	0.46678 (10)	0.38805 (11)	0.4341 (2)	0.0861 (8)	
H10A	0.4619	0.4096	0.5017	0.129*	
H10B	0.4910	0.3544	0.4475	0.129*	
H10C	0.4291	0.3755	0.4076	0.129*	
C11	0.19775 (8)	0.55590 (9)	0.01807 (17)	0.0588 (6)	
H11A	0.1818	0.5947	0.0115	0.088*	
H11B	0.1674	0.5293	0.0408	0.088*	
H11C	0.2132	0.5437	−0.0522	0.088*	
C12	0.43929 (8)	0.56124 (7)	0.05932 (14)	0.0401 (4)	
C13	0.39064 (9)	0.65416 (8)	0.00759 (16)	0.0529 (5)	
H13A	0.3761	0.6366	−0.0593	0.079*	
H13B	0.4288	0.6705	−0.0057	0.079*	
H13C	0.3644	0.6847	0.0308	0.079*	

### Atomic displacement parameters (Å^2^)

**Table:**

	*U*^11^	*U*^22^	*U*^33^	*U*^12^	*U*^13^	*U*^23^
N1	0.0401 (9)	0.0565 (10)	0.0546 (10)	0.0059 (8)	−0.0052 (7)	0.0066 (8)
N2	0.0373 (9)	0.0459 (9)	0.0457 (9)	0.0019 (7)	0.0041 (7)	−0.0026 (7)
N3	0.0342 (8)	0.0456 (9)	0.0655 (11)	0.0027 (7)	0.0014 (7)	−0.0195 (8)
O1	0.0444 (8)	0.0558 (8)	0.0661 (9)	0.0102 (6)	−0.0121 (6)	0.0019 (7)
O2	0.0574 (9)	0.0617 (10)	0.0890 (11)	−0.0071 (7)	−0.0152 (8)	0.0097 (8)
O3	0.0346 (7)	0.0627 (8)	0.0529 (8)	−0.0010 (6)	0.0000 (6)	0.0073 (6)
O4	0.0338 (7)	0.0607 (9)	0.0766 (10)	0.0034 (6)	−0.0029 (6)	−0.0254 (7)
C1	0.0360 (11)	0.0753 (14)	0.0560 (13)	0.0088 (9)	0.0019 (9)	0.0136 (10)
C2	0.0345 (10)	0.0412 (10)	0.0442 (10)	0.0093 (8)	0.0030 (8)	−0.0041 (8)
C3	0.0398 (10)	0.0369 (10)	0.0447 (10)	0.0042 (8)	0.0025 (8)	−0.0042 (8)
C4	0.0525 (12)	0.0423 (11)	0.0559 (12)	0.0054 (9)	−0.0005 (9)	−0.0119 (9)
C5	0.0593 (13)	0.0571 (13)	0.0509 (12)	0.0100 (10)	−0.0094 (10)	−0.0106 (10)
C6	0.0433 (12)	0.0622 (14)	0.0453 (12)	0.0027 (10)	−0.0009 (9)	0.0055 (10)
C7	0.0512 (12)	0.0570 (13)	0.0549 (13)	0.0139 (9)	0.0011 (10)	0.0058 (10)
C8	0.0959 (19)	0.0966 (19)	0.0744 (17)	0.0334 (15)	−0.0017 (14)	−0.0162 (14)
C9	0.0470 (13)	0.0821 (16)	0.0837 (17)	0.0128 (11)	−0.0020 (11)	0.0093 (13)
C10	0.0726 (16)	0.0894 (18)	0.0962 (19)	0.0002 (13)	−0.0024 (14)	0.0364 (15)
C11	0.0409 (11)	0.0714 (14)	0.0641 (13)	−0.0025 (9)	−0.0097 (10)	0.0085 (11)
C12	0.0345 (10)	0.0393 (10)	0.0467 (11)	−0.0006 (8)	0.0024 (8)	−0.0035 (8)
C13	0.0551 (12)	0.0464 (11)	0.0571 (12)	0.0021 (9)	0.0057 (9)	0.0021 (9)

### Geometric parameters (Å, °)

**Table:**

N1—C6	1.352 (2)	C4—H4B	0.9700
N1—C1	1.444 (2)	C5—H5A	0.9700
N1—C5	1.453 (2)	C5—H5B	0.9700
N2—C2	1.272 (2)	C7—C10	1.509 (3)
N2—O3	1.4217 (17)	C7—C9	1.509 (3)
N3—C12	1.323 (2)	C7—C8	1.509 (3)
N3—H3A	0.8600	C8—H8A	0.9600
N3—H3B	0.8600	C8—H8B	0.9600
O1—C6	1.339 (2)	C8—H8C	0.9600
O1—C7	1.465 (2)	C9—H9A	0.9600
O2—C6	1.213 (2)	C9—H9B	0.9600
O3—C11	1.427 (2)	C9—H9C	0.9600
O4—C12	1.2308 (19)	C10—H10A	0.9600
C1—C2	1.497 (2)	C10—H10B	0.9600
C1—H1A	0.9700	C10—H10C	0.9600
C1—H1B	0.9700	C11—H11A	0.9600
C2—C3	1.515 (2)	C11—H11B	0.9600
C3—C13	1.531 (2)	C11—H11C	0.9600
C3—C4	1.533 (2)	C13—H13A	0.9600
C3—C12	1.539 (2)	C13—H13B	0.9600
C4—C5	1.526 (3)	C13—H13C	0.9600
C4—H4A	0.9700		
			
C6—N1—C1	118.66 (17)	O1—C7—C10	109.46 (16)
C6—N1—C5	125.56 (16)	O1—C7—C9	101.60 (15)
C1—N1—C5	115.73 (16)	C10—C7—C9	110.25 (18)
C2—N2—O3	109.33 (14)	O1—C7—C8	109.82 (17)
C12—N3—H3A	120.0	C10—C7—C8	113.1 (2)
C12—N3—H3B	120.0	C9—C7—C8	111.98 (17)
H3A—N3—H3B	120.0	C7—C8—H8A	109.5
C6—O1—C7	121.90 (15)	C7—C8—H8B	109.5
N2—O3—C11	110.11 (13)	H8A—C8—H8B	109.5
N1—C1—C2	110.52 (15)	C7—C8—H8C	109.5
N1—C1—H1A	109.5	H8A—C8—H8C	109.5
C2—C1—H1A	109.5	H8B—C8—H8C	109.5
N1—C1—H1B	109.5	C7—C9—H9A	109.5
C2—C1—H1B	109.5	C7—C9—H9B	109.5
H1A—C1—H1B	108.1	H9A—C9—H9B	109.5
N2—C2—C1	123.83 (16)	C7—C9—H9C	109.5
N2—C2—C3	119.79 (16)	H9A—C9—H9C	109.5
C1—C2—C3	116.37 (15)	H9B—C9—H9C	109.5
C2—C3—C13	112.63 (14)	C7—C10—H10A	109.5
C2—C3—C4	105.95 (14)	C7—C10—H10B	109.5
C13—C3—C4	110.71 (14)	H10A—C10—H10B	109.5
C2—C3—C12	110.73 (13)	C7—C10—H10C	109.5
C13—C3—C12	107.33 (14)	H10A—C10—H10C	109.5
C4—C3—C12	109.49 (14)	H10B—C10—H10C	109.5
C5—C4—C3	113.25 (14)	O3—C11—H11A	109.5
C5—C4—H4A	108.9	O3—C11—H11B	109.5
C3—C4—H4A	108.9	H11A—C11—H11B	109.5
C5—C4—H4B	108.9	O3—C11—H11C	109.5
C3—C4—H4B	108.9	H11A—C11—H11C	109.5
H4A—C4—H4B	107.7	H11B—C11—H11C	109.5
N1—C5—C4	110.80 (15)	O4—C12—N3	122.35 (15)
N1—C5—H5A	109.5	O4—C12—C3	120.75 (15)
C4—C5—H5A	109.5	N3—C12—C3	116.84 (15)
N1—C5—H5B	109.5	C3—C13—H13A	109.5
C4—C5—H5B	109.5	C3—C13—H13B	109.5
H5A—C5—H5B	108.1	H13A—C13—H13B	109.5
O2—C6—O1	125.26 (19)	C3—C13—H13C	109.5
O2—C6—N1	124.14 (18)	H13A—C13—H13C	109.5
O1—C6—N1	110.60 (17)	H13B—C13—H13C	109.5
			
C2—N2—O3—C11	−173.54 (15)	C1—N1—C5—C4	−40.4 (2)
C6—N1—C1—C2	−118.26 (19)	C3—C4—C5—N1	−20.9 (2)
C5—N1—C1—C2	59.1 (2)	C7—O1—C6—O2	8.3 (3)
O3—N2—C2—C1	0.3 (2)	C7—O1—C6—N1	−172.16 (15)
O3—N2—C2—C3	−178.79 (13)	C1—N1—C6—O2	0.0 (3)
N1—C1—C2—N2	167.35 (16)	C5—N1—C6—O2	−177.08 (19)
N1—C1—C2—C3	−13.5 (2)	C1—N1—C6—O1	−179.54 (15)
N2—C2—C3—C13	16.4 (2)	C5—N1—C6—O1	3.4 (3)
C1—C2—C3—C13	−162.72 (16)	C6—O1—C7—C10	−68.6 (2)
N2—C2—C3—C4	137.60 (16)	C6—O1—C7—C9	174.80 (16)
C1—C2—C3—C4	−41.54 (19)	C6—O1—C7—C8	56.1 (2)
N2—C2—C3—C12	−103.77 (18)	C2—C3—C12—O4	−142.74 (17)
C1—C2—C3—C12	77.10 (19)	C13—C3—C12—O4	93.96 (19)
C2—C3—C4—C5	59.91 (19)	C4—C3—C12—O4	−26.3 (2)
C13—C3—C4—C5	−177.69 (15)	C2—C3—C12—N3	40.0 (2)
C12—C3—C4—C5	−59.54 (19)	C13—C3—C12—N3	−83.25 (19)
C6—N1—C5—C4	136.72 (18)	C4—C3—C12—N3	156.51 (15)

### Hydrogen-bond geometry (Å, °)

**Table:**

*D*—H···*A*	*D*—H	H···*A*	*D*···*A*	*D*—H···*A*
N3—H3A···O4^i^	0.86	2.11	2.9607 (18)	173
N3—H3B···O3^ii^	0.86	2.34	3.1334 (19)	153

Symmetry codes: (i) −*x*+1, −*y*+1, −*z*; (ii) *y*−1/4, −*x*+3/4, *z*−1/4.
